# Supplementary material for: Direct imaging of delayed magneto-dynamic modes induced by surface acoustic waves
Source: Nat Commun. 2017 Sep 1;8:407. doi: 10.1038/s41467-017-00456-0 (PMC5581333; doi:10.1038/s41467-017-00456-0)
Supplement: Supplementary file 1 — Supplementary Information [file 41467_2017_456_MOESM1_ESM.pdf]

File name: Supplementary Information
Description: Supplementary Figures, Supplementary Notes and Supplementary References.

File name: Supplementary Movie 1
Description: The SAWs produce a contrast in the PEEM images because the piezoelectric voltage associated with the strain wave shifts the energy of the secondary electrons. Thus, bright and dark stripe lines with the periodicity of the SAW excitation (wavelength, 8 μm) appear in the PEEM images. We compiled a video with PEEM images corresponding to different electron kinetic energy (that were controlled in our detector). Note the contrast inversion during the scan corresponding to the ranges highlighted in Figure 2b.

File name: Supplementary Movie 2
Description: We recorded PEEM images with a SAW frequency having a small (sub-Hz) detuning with respect to the synchrotron bunch frequency to confirm the SAW propagation direction by direct observation of the displacement of the stripes in the PEEM images. This video shows both positive (left) and negative (right) 0.01 Hz detuning that confirms the wave propagation.

File name: Supplementary Movie 3
Description: PEEM (left) and PEEM/XMCD (right) images are combined in a video that shows the simultaneous evolution of the piezoelectric voltage and the magnetic domain configuration in the hybrid sample of LiNbO3 with Ni squares.

File name: Peer Review File
Description:

Supplementary Note 1: Micromagnetics Results

We modeled the dynamic anisotropy variations in the Ni nanostructures with micromagnetic simulations using the open-source MuMax3 code[1] on a graphics card with 2048 processing cores. Simulation parameters are reported in the Methods section and a simplified code is appended at the end of this document.

We can estimate the induced anisotropy of a given magnetic-domain configuration and thus we can translate the variations observed in the experiments into variations of the magnetic anisotropy. Magnetic domain configurations corresponding to different uniaxial anisotropies are shown in Supplementary Figure 1. The top panels show magnetic-domain configuration on a Ni square 2×2 μm^2 considering that the anisotropy axis is along the square sides whereas the lower panels present the case where anisotropy axis is along the diagonal of the square.

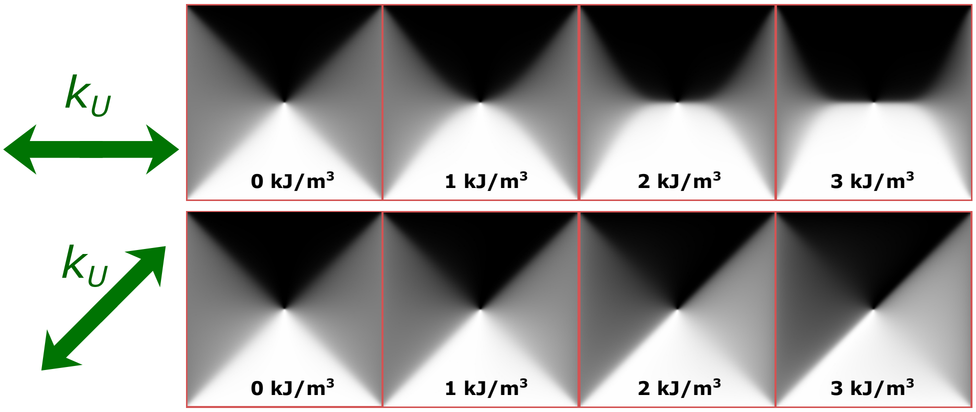

Supplementary Figure 1. **Magnetic domain configurations corresponding to different uniaxial anisotropies**. Top panels show magnetic-domain configuration on a Ni square 2×2 μm^2 with 20 nanometer in thickness, considering that the anisotropy axis is along the square sides. Lower panels present the case where anisotropy axis is along square diagonal on the same micrometric structures. Values of anisotropy, $k_{\rm U}$, from 0 to 3 KJm^{-3} are plotted.

The studied magnetic nanostructures have internal resonances in the magnetic domains, in the domain walls, and even in the vortex formed in the center of the Landau flux closure states. Raabe *et al.*[2] experimentally identified three different dynamic processes—with different timescales—in a Ni micrometric squares under a short magnetic pulse; i) precessional

motion within the magnetic domain, ii) domain-wall resonance and iii) vortex motion. In our experiment we studied two configurations that precisely can serve to separate the dynamics involving domain-wall motion (square sides aligned with the SAW) from dynamics that only involves magnetization rotation within a domain (square diagonal aligned with the SAW).

We note that effect of magnetization on the strain wave is not considered in this model. We estimated (see next section) that the effect of the magnetization changes on the strain are small and thus can be neglected in our system. However, models including strain and magnetic forces might be used to obtain more accurate results[3,4]. We have introduced in the simulations a time varying anisotropy with a fixed wavelength $\lambda_{\mathrm{SAW}} = 8$ μm and measured the magnetic response of the Ni squares under different frequencies of the oscillating anisotropy.

In order to quantify the dynamic state of the Ni squares at each frequency we calculated the same quantities shown for the experimental results: *i)* relative black and white area for the configuration with SAW along square sides and *ii)* difference of intensity within the two gray domains for the SAW along square diagonals. Supplementary Figure 2 plots the evolution of the dynamic state of Ni squares with amplitude (showing real and imaginary parts) and phase as a function of frequency. We can see in Supplementary Figure 2 how the phase increases with frequency for the two configurations as the system goes through the resonances: for the configuration with SAW aligned with square sides, the phase begins to increase with the domain-wall resonance (see Supplementary Figure. 2a) whereas for the configuration of SAW aligned with square diagonals, there is no domain-wall resonance and the phase begins to increase at a higher frequency (see Supplementary Figure 2b). We have introduced markers in the lower panels of Supplementary Figure 2 at $f = 500$ MHz to show the phase value for the two configurations (30 deg for SAW along square sides and 3 deg for SAW along square diagonals). We note that resonance frequencies (and thus phase values) are dependent on the material parameters. We have performed extensive micromagnetic simulations with different values of A (exchange constant), M_{s} (saturation magnetization) and α (damping parameter) and found the following overall effects on the domain resonance (DR) and domain-wall resonance (DWR): *i)* increasing α increases the width of the resonances, *ii)* increasing M_{s} shifts the DR towards higher frequencies and barely moves the DWR and *iii)* increasing A shifts the DWR towards higher frequencies and barely changes the DR. We also note here that frequencies below the resonance produce

a considerable change in the magnetization state but with phase shift (delay) that increases as we approach the resonance.

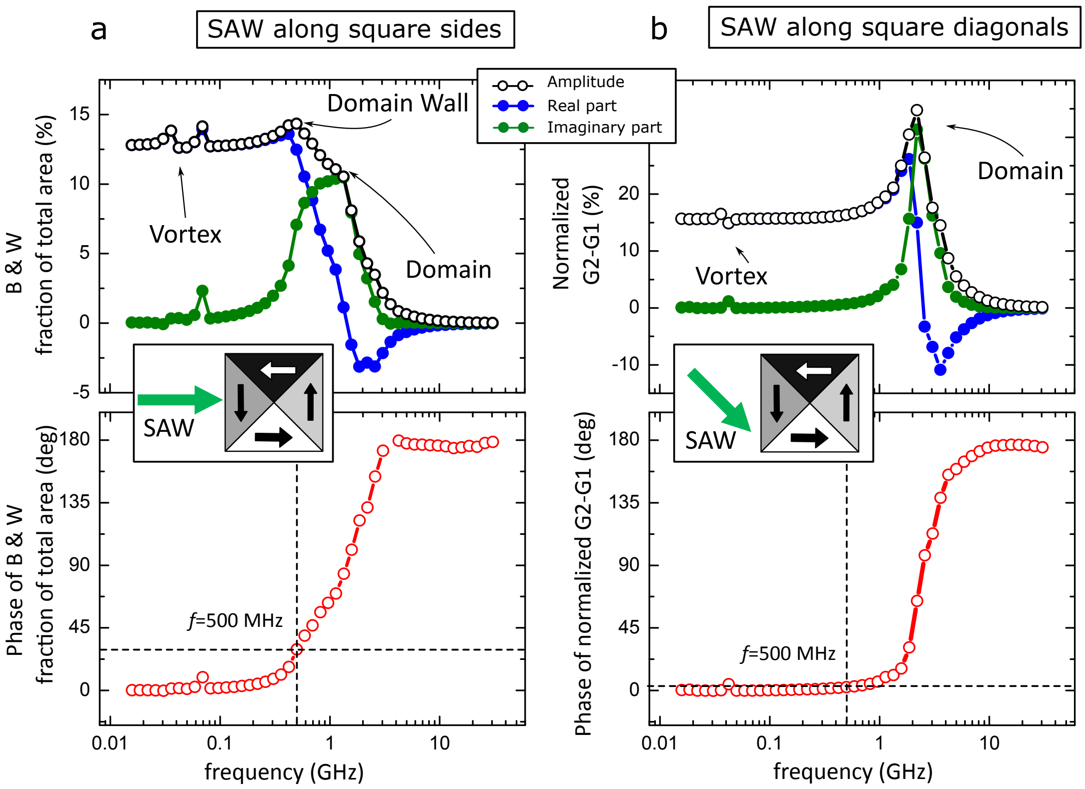

Supplementary Figure 2. **Magnetic response in Ni squares caused by SAW**. Amplitude, in phase (real), and out of phase (imaginary) parts (and phase in the lower panels) of the magnetic response of the system of 2×2 µm Nickel squares to an oscillating anisotropy as a function of the frequency. Two configurations are plotted: in a) the varying anisotropy axis is along the square sides, in b) the varying anisotropy axis is along the square diagonals. We plotted for each configuration the same quantities we analyzed for the experimental data: in a) we plotted the variation (in %) of the fraction area of black and white domains and in b), the variation (in %) of the normalized difference between gray intensities between the two gray domains. Dashed lines (black) in the lower panels mark the frequency of $f = 500$ MHz, which corresponds to the frequency used in the experiments. For the configuration of panels a the phase shift corresponds to 30 deg whereas the configuration of panels b results in almost no delay ($\ll 3$ deg).

Supplementary Note 2: Strain induced by the magnetic modulation

We have calculated the strain caused by the magnetization changes in our samples in both configurations *(a)* varying anisotropy along square sides and *(b)* varying anisotropy along square diagonal). Strain is proportional to the magnetization with the magneto-mechanical coupling tensor λ (which we have taken for Nickel from[4]). If we consider the strain variation caused in our sample between the two extreme magnetization states (given by the two extremes of the SAW), we find that the maximum strain difference is 6×10^{-5} (for both S_{xx} and S_{yy}, not at the same time), which is about 13% of the strain we are applying (4.5×10^{-4}). Supplementary Figure 3 shows the strain variation induced in nickel squares due to extremal changes in magnetization: the two magnetization states corresponding to the maximum and minimum values of anisotropy are plotted in the top panels in both configurations (a) for SAW propagation aligned with the squares side and (b) SAW propagation aligned with the squares diagonal. Such a calculation would be valid for a sample with no boundary conditions but our sample is clamped to the $LiNbO_3$ surface and thus the real change of strain or deformation could be expected to be much lower. We thus consider that our model still describes accurately the presented experiment.

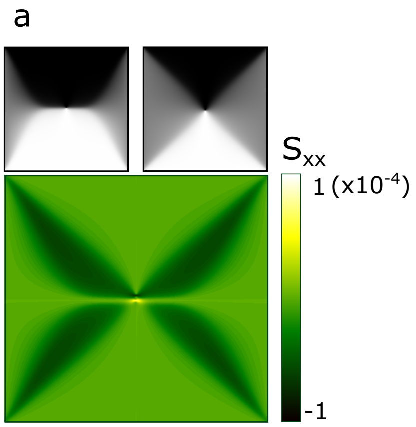

Supplementary Figure 3. **Strain maps in Ni squares**. Strain variation induced in a 2 × 2 μm nickel square due to extremal changes in magnetization. The two magnetization states corresponding to the maximum and minimum values of anisotropy are plotted in the top panels in both configurations (a) for SAW propagation aligned with the squares side and (b) SAW propagation aligned with the squares diagonal. The bottom panel corresponds to the component S_{xx} of the strain tensor caused by the magnetization difference. It has a maximum value of $\pm6\times10^{-5}$ in the darkest/brightest areas.

Supplementary Note 3: Micromagnetics code

```
// mumax3 is a GPU-accelerated micromagnetic simulation open-source software
// developed at the DyNaMat group of Prof. Van Waeyenberge at Ghent University.
// The mumax3 code is written and maintained by Arne Vansteenkiste.

//GRID
CellSize:=4.e-9
NumCells:=512
SetGridSize(NumCells, NumCells, 1)
SetCellSize(CellSize, CellSize, 20.e-9)

Setgeom(universe())

//MATERIAL PARAMETERS FOR STANDARD Ni
Msat=490e3
Aex=5e-12
Alpha = 0.03

//INITIAL MAGNETIZATION STATE
m = vortex(1,1)

//REGIONS
MaxRegion:=200
CellsPerRegion:=NumCells/MaxRegion
RegionWidth:=CellsPerRegion*CellSize
SampleCenter:= CellSize*NumCells/2.

for i:=0; i<=MaxRegion; i++ {
defregion(i, xrange(i*RegionWidth-SampleCenter,1))
}

//DEFINING ANISOTROPY VECTOR
for i:=0; i<=MaxRegion; i++{
AnisU.SetRegion(i,vector(1.,0.,0.))
}

//DEFINING ANISOTROPY CONSTANT
 freq:=500000. //SAW freq
 Kuav:=1.2e3  //Nominal Anisotropy
 Kumod:=1.e3
 Lambd:=4.     //in sample width units
for i:=0; i<=MaxRegion; i++{
ku1.SetRegion(i, Kuav + Kumod * cos( 2*pi*((i* 1/MaxRegion -0.5)/Lambd - freq*t)))
}

relax()
run(20e-9)
```

SUPPLEMENTARY REFERENCES

[1] Vansteenkiste, A. *et al.* Lattice-strain control of the activity in dealloyed core-shell fuel cell catalysts. AIP Adv. **2**, 107133(2014)

[2] Raabe, J. *et al.* Quantitative Analysis of Magnetic Excitations in Landau Flux-Closure Structures Using Synchrotron-Radiation Microscopy. *Phys. Rev. Lett.* **94**, 217204 (2005)

[3] Sohn, H. *et al.* Electrically Driven Magnetic Domain Wall Rotation in Multiferroic Heterostructures to Manipulate Suspended On-Chip Magnetic Particles. *ACS Nano* **9** 4814-4826 (2015)

[4] Liang, C.-Y. *et al.* Modeling of magnetoelastic nanostructures with a fully coupled mechanical-micromagnetic model. *Nanotechnology* **25**, 435701 (2014)
